# Cell Cycle Inhibition without Disruption of Neurogenesis Is a Strategy for Treatment of Aberrant Cell Cycle Diseases: An Update

**DOI:** 10.1100/2012/491737

**Published:** 2012-04-01

**Authors:** Da-Zhi Liu, Bradley P. Ander

**Affiliations:** Department of Neurology and the MIND Institute, University of California at Davis, Sacramento, CA 95817, USA

## Abstract

Since publishing our earlier report describing a strategy for the treatment of central nervous system (CNS) diseases by inhibiting the cell cycle and without disrupting neurogenesis (Liu et al. 2010), we now update and extend this strategy to applications in the treatment of cancers as well. Here, we put forth the concept of “aberrant cell cycle diseases” to include both cancer and CNS diseases, the two unrelated disease types on the surface, by focusing on a common mechanism in each aberrant cell cycle reentry. In this paper, we also summarize the pharmacological approaches that interfere with classical cell cycle molecules and mitogenic pathways to block the cell cycle of tumor cells (in treatment of cancer) as well as to block the cell cycle of neurons (in treatment of CNS diseases). Since cell cycle inhibition can also block proliferation of neural progenitor cells (NPCs) and thus impair brain neurogenesis leading to cognitive deficits, we propose that future strategies aimed at cell cycle inhibition in treatment of aberrant cell cycle diseases (i.e., cancers or CNS diseases) should be designed with consideration of the important side effects on normal neurogenesis and cognition.

## 1. Introduction

The cell cycle is an irreversible, ordered set of events that normally leads to cellular division [1–5]. The release of cells from a quiescent state (G0) results in their entry into the first gap phase (G1), during which the cells prepare for DNA replication in the synthetic phase (S). This is followed by the second gap phase (G2) and mitosis phase (M). After the cell has split into its two daughter cells, the new cells enter either G1 or G0. Tumors usually originate from adult tissues, in which the majority of cells are in the G0 quiescent phase [4]. Mature neurons normally maintain themselves in G0 resting phase. These facts suggest that the cells that go on to form tumors and mature neurons share a common G0 state of quiescence.

Since cell cycle is irreversible, this raises a possibility that irreversible cell cycle reentry mediates the irreversible neuronal death that mirrors the irreversible progression of some central nervous system (CNS) diseases, such as Alzheimer's disease (AD). If this is true, it will partially explain why AD is incurable once even early AD symptoms occur, for the early AD symptoms may indicate that the neurons have reentered the cell cycle that ends up leading to neuronal death and AD progression. Thus, the best strategy in treatment of CNS diseases is to prevent cell cycle re-entry at the early stage before neurons leave the G0 phase at all, since even the mere entrance into the initial cell cycle may lead to unavoidable neuronal death.

Since re-entry into the cell cycle by tumor cells or neurons has been associated with many tumor or CNS diseases and linked to uncontrolled cell proliferation (in cancer) or neuronal death (in CNS diseases), cell cycle inhibition strategies are of interest in the treatment of both tumor and CNS diseases. For instance, the cell cycle inhibitors, such as cyclin-dependent kinase (CDK) inhibitors, have been widely studied as cancer therapeutics. They have been used to inhibit growth of several types of tumor cells in numerous preclinical studies, both *in vitro* and *in vivo* [6–12]. Several cell cycle inhibitors have advanced to human clinical trials for evaluation as a treatment for a broad range of solid tumors and hematological malignancies such as chronic lymphocytic leukemia (CLL) [13–17]. Though no clinical trials of the cell cycle inhibitors are reported in the treatment of CNS diseases, preclinical experiments demonstrate that the cell cycle inhibitors improve behavioral outcomes and increase neuronal survival in a series of CNS disease models [18–33].

Cell cycle inhibition kills tumor cells (in treatment of cancer) or protects mature neurons from death (in treatment of CNS diseases), whereas this can also block proliferation of neural progenitor cells (NPCs) and thus impair brain neurogenesis leading to cognitive deficits in the patients of cancer and CNS diseases [1]. Since the presence of cognitive deficits is a major factor markedly affecting quality of life of these patients, the cell cycle inhibition strategy in treatment of cancer and CNS diseases should consider the consequences on other cell types that can be affected, such as NPCs.

As a way to describe the two seemingly different disease types (i.e., cancer and CNS diseases) that share the common mechanism of cell cycle re-entry, we propose a broader term of “aberrant cell cycle diseases”—one which includes not only cancers but also CNS diseases. A detailed description of how the cell cycle re-entry, at least in part, underlies cancers and CNS diseases follows before we discuss the pharmacological approaches that have been examined in therapeutic treatment of the two disease types.

## 2. Aberrant Cell Cycle Diseases: Cancers and CNS Diseases

Cancers and CNS diseases are two major threats to human health. Epidemiological studies show that patients with CNS disease, such as Alzheimer's disease (AD), Parkinson's disease (PD), Huntington's disease (HD), and multiple sclerosis (MS), have a significantly lower risk of most cancers [34–38]. The reverse correlations also hold true: cancer survivors have a significantly lower risk of developing some of these CNS diseases. However, there are exceptions: Parkinson's patients have an increased risk for melanoma [39–44], autism patients have an increased risk for breast cancer [45], and Fragile X Syndrome patients have increased risk for lip cancer [46]. No matter what associations underlie certain cancers and CNS diseases, these correlative studies have raised an interesting question: what associated processes or mechanisms do dying neurons and growing tumor cells have in common?

Aberrant cell cycle is the hallmark of many tumor cells in cancers [47–49] and is also observed in postmortem and/or animal studies of dying neurons in a series of CNS diseases, such as AD, PD, stroke, epilepsy, cerebral hypoxia-ischemia, amyotrophic lateral sclerosis (ALS), traumatic brain injury (TBI), among others [18–33, 50–64]. Although tumor cells undergo uncontrolled proliferation, many tumors originate from adult tissues in which the majority of cells are in the G0 quiescent phase [4]. Similarly, mature neurons stay in G0 quiescent phase in normal physiological conditions, but do reenter the cell cycle irregularly (and die) in certain pathological conditions.

Thus, the cells that go on to form tumors and healthy neurons share a common G0 state of quiescence. However, if tumor cells re-enter the cell cycle, they survive and often proliferate, whereas mature neurons will die. Therefore, cell cycle inhibition can be applied not only to kill tumor cells (in treatment of cancer), but also to protect neurons from death (in treatment of CNS diseases). This is strongly supported by the findings that mutation of PARK2, a tumor suppressor gene, results not only in neuronal death in Parkinson's disease, but also in tumor cell proliferation in glioblastoma and other human cancer [65]. Consistently, cell cycle inhibition mot only promotes the death of naive PC12 (pheochromocytoma) tumor cells, but also prevents the death of nerve growth factor- (NGF-) differentiated PC12 neuronal cells [66, 67].

## 3. Cell Cycle and “Expanded Cell Cycle”

Classically, the proteins and regulators of the cell cycle include CDKs, cyclins, CDK inhibitors, and CDK substrates. However, the cell cycle inhibition strategies that target on these molecules lack specificity for their target tumor cells or dying neurons and thus interfere the normal biological processes performed by other cell types, since the core cell-cycle-associated molecules are often highly conserved throughout eukaryotes, thus we proposed an “expanded cell cycle” to include not only the core cell cycle molecules mentioned above, but also the mitogenic molecules and the signaling pathways that interact with them. Under specific environmental and/or pathological conditions, such as exposure of tobacco smoke, benzene, ultraviolet B radiation, and/or enhancement of mitogenic molecules (i.e., thrombin, growth factors, amyloid beta, etc.), activation of specific pathways to mediate abnormal cell cycle re-entry may arise and thus trigger tumorigenesis of normal cells and/or death of neurons. It is always impossible to prove exactly what caused a cancer or CNS disease in any individual, because most of these diseases have multivariate causes. However, the various causes seem to result in a common outcome—cell cycle re-entry, mediated by several common mitogenic pathways. The main mitogenic pathways include (1) FAK/Src/Ras/Raf/MEK1, 2/ERK1, 2 → cell cycle re-entry [68–72]; (2) Ras/Rac1/MEK3, 6/P38 → cell cycle re-entry [73, 74]; (3) PLC/IP3/PKC/JNK → cell cycle re-entry [75, 76]; (4) PI3K/Akt/mTOR/Tau → cell cycle re-entry [60, 77, 78]; (5) JAK/STAT → cell cycle re-entry [79, 80]. In addition, many molecules, including ROS, PGE_2_, NO, and Ca^2+^, can directly or indirectly participate in the main mitogenic signaling pathways [81–86]. The idea of an “expanded cell cycle” provides a wider view encompassing a broad range of molecules representing potential targets and thus approaches that can serve as treatments for cancer and CNS diseases—all sharing the common outcome of cell cycle inhibition.

## 4. Pharmacological Approaches Interfering with the “Expanded Cell Cycle” in Treatment of Aberrant Cell Cycle Diseases

In theory any part of the “expanded cell cycle” could act as a potential target for drug discovery. For example, thrombin activation is substantially increased in cancers [87–89] and CNS diseases (i.e., AD, stroke). Thrombin may then go on to activate Src kinases [90, 91]. Src kinases will activate MAPK which will activate CDK4/cyclinD complexes and promote cell cycle re-entry [71, 91, 92]. Thus, these molecules (thrombin, Src kinases, and MAPK), while not considered traditional components of the cell cycle, would all be considered part of the “expanded cell cycle.” Similarly, other protein kinases (including JAK, Akt, PKC, JNK, ERK, GSK-3*β*.) are also important molecules in the mitogenic pathways leading to neuronal cell cycle re-entry. Most targets mentioned above have been examined in cancer therapies as well as in CNS diseases. Pharmacological approaches based on those targets include traditional cell cycle inhibitors (CDK inhibitors), antioxidants, NMDA-receptor modulators, i/eNOS inhibitors, COX-2 inhibitors, protein kinase inhibitors, and others (Table 1).

## 5. Cognitive Side Effects of Clinic Therapies for Aberrant Cell Cycle Diseases

In treatment of peripheral or brain cancers, surgical removal of the tumor is recommended whenever possible. Anticancer medications (chemotherapy, CT) may be prescribed, as well as radiation therapy (RT). CT- or RT is cytotoxic, not only slowing down or killing rapidly dividing cells and producing many different types of DNA damage [93], but detrimentally leading to damage of normal tissue, especially of fast-growing healthy cells, including neural progenitor cells (NPCs), and also red and white blood cells [94, 95]. Recent reports show that CT results in cognitive side effects in extracranial cancer (i.e., breast cancer, prostate, etc.) patients [96–98], and CT or RT has also been associated with neurogenesis impairment and long-term cognitive deficits in brain cancer patients [99–105]. The main idea that is thought to contribute to the CT or RT-induced cognitive decline is that these treatments block proliferation of NPCs in the hippocampal and periventricular zones, which cannot be repopulated as healthy cells die. This leads to cognitive decline, since the NPCs help maintain neurogenesis [106–109], repair damage from brain injury [105, 109–118], and are important in cognition [119–121].

Besides the CT and RT mentioned above in treatment of cancers, some pharmacological approaches that interfere with classical cell cycle molecules or mitogenic pathways have been examined in cancer and CNS disease therapies (Table 1). Therapies directed at any component inhibiting the cell cycle must be as specific as possible considering cell cycle re-entry contributes to the proliferation of tumor cells, the death of mature neurons, and the genesis of NPCs in adult brain. Therefore, any therapeutics that prevent tumor growth and/or neuronal death by blocking cell cycle re-entry may have limited benefit because they may impair neurogenesis and thus lead to cognitive side effects. This may provide at least a partial explanation for the questionable efficacy of some currently approved drugs, such as the NMDA receptor modulator Memantine, in the clinical treatment of AD [122], since NMDA receptor inhibition has been shown to block progenitor cell proliferation and lead to impaired neurogenesis [123].

The cognitive side effects may be explained by the fact that current cell cycle inhibition strategies are not cell specific and also block the proliferation of important brain progenitor cells, thus impairing adult brain neurogenesis. If drugs that block the cell cycle are used to kill tumor cells (in treatment of cancer) and/or help protect neurons (in treatment of CNS diseases), it is likely that compounds would need to directly (or indirectly) block tumor and neuronal cell cycle re-entry and yet not affect the ongoing process of neurogenesis. This will only be possible if the signaling mechanisms are different in NPCs that divide in the adult brain, versus tumor cells and neurons that re-enter the cell cycle irregularly.

## 6. Conclusions

Cancer and CNS diseases, two seemingly different disease types, at least in part share the common molecular pathology of cell cycle re-entry. With this knowledge in mind, novel insights into cell cycle inhibition strategies to be used in treatment of the “aberrant cell cycle diseases” may be made. Future studies aimed at better understanding the respective cell cycle pathways of tumor cells, neurons, and NPCs are probably necessary before choosing the best drug targets for treating certain “aberrant cell cycle diseases” so as to consider the most effective benefits to the patient without causing indirect harm in related, but different systems.

## Figures and Tables

**Table 1:** Pharmacological approaches interfering with mitogenic molecules and signaling pathways of the “expanded cell cycle” in treatments of cancer and CNS diseases.

	Treatments			
Targets	Cancers		CNS diseases	
	Agents	Stages	Agents	Stages
CDK inhibitors	Flavopiridol, Indisulam, AZD5438 SNS-032 Bryostatin-1 Seliciclib PD 0332991 SCH 727965 UCN-01, Roscovitine, AT7519	Clinical trials in a broad range of solid tumors and chronic lymphocytic leukemia (CLL) [13–16].	Flavopiridol Olomoucine Roscovitine Quinazolines	Experimental trials in AD [18–22], PD [21], stroke [23, 24], TBI [25, 26], SCI [27, 28], excitotoxic stress [29–32], optic nerve transaction [33].

Antioxidants	Isoliquiritigenin	Experimental trials in prostate cancer [124].	Edaravone NXY-059 Coenzyme Q10 Vitamin E Melatonin Trolox SOD NAC PBN	Clinical use in stroke in Asia [125, 126], Clinical trials in stroke [126], AD [127–129], ALS [130–132]. Experimental trials in ALS [133], PD [134], SCI [135].

i/eNOS inhibitors	L-NAME AMT NPA	Experimental trials in prostate cancer [136].	L-NAME AMT NPA	Experimental trials in ICH [137], SCI [135].

Cox-2 inhibitors	Celecoxib Rofecoxib NS-398	Clinical trials in bladder cancer [138], lung cancer [139, 140], head and neck cancer [141], pancreatic cancer [142], prostate cancer [143], breast cancer [144], colorectal cancer [145]. Experimental trials in pancreatic cancer [146], prostate cancer [147], esophageal cancer [148], colon cancer [149].	Celecoxib Rofecoxib NS-398	Clinical trials in AD [150, 151]. Experimental trials in ICH [137].

Ca^2+^ channel blockers	KYS05090	Experimental trials in cancers [152].	Flunarizine	Clinical trials in stroke [153].

Glutamatergic modulators	MK-801	Experimental trials in breast cancer [154], brain tumors [155].	Riluzole Ceftriaxone Talampanel MK-801 NBQX	Clinical trials in ALS [156]. Experimental trials in TBI [157, 158], SCI [159], ICH [160], stroke [161].

NMDA-receptor modulators	AP5 Memantine	Experimental trials in breast cancer [154], gastric cancer [162].	Memantine	Clinical use in AD [163–166]. Clinical trials in ALS [156]. Off-label use in psychiatric disorders [167]. Experimental trials in TBI [168], ICH [169], stroke [170],

Thrombin inhibitors	Heparin	Experimental trials in lung cancer [171].	Heparin Hirudin	Experimental trials in ischemic stroke [172], ICH [118, 173, 174].

Thrombin receptor-1 antagonist	RWJ-58259 SCH-79797	Experimental trials in colon cancer [175].	BMS-200261	Experimental trials in stroke [176]; PD [177].

Ras inhibitors	Lovastatin FTS	Clinical trials in neurofibroma [178], head and neck cancer [179]. Experimental trials in neurofibroma [180], liver cancer [181], ovarian cancer [182], breast cancer [183], prostate cancer [184], lung cancer [185].	Lovastatin Exoenzyme	Clinical trials in acute ischemic stroke [186, 187]. Experimental trials in motor neuron disorders [188, 189].

Src Inhibitors	KX-01 Dasatinib PP1 PP2 Saracatinib	Clinical trials in breast cancer [190–192]. Experimental trials in breast cancer [193], lung cancer [194, 195], cervical cancer [196], renal cancer [197], prostate cancer [198].	PP1 PP2	Experimental trials in ICH [91, 199], AD [70].

JAK/Stat Inhibitors	EGCG WP-1034	Experimental trials in prostate cancer [200], breast cancer [201], lung cancer [202], pancreatic cancer [203], acute myeloid leukemia [204].	EGCG	Experimental trials in AD, PD, HIV associated Dementia, multiple sclerosis (MS), ALS, or Pick's Disease [79].

GSK-3*β* inhibitors	Lithium SB 415286 SB 216763 AR-A014418	Experimental trials in colon cancer [205], neuroblastoma [206].	L803-mt Lithium Kenpaullone Indirubin SB 216763 SB 415286	Experimental trials in AD [207–212], PD [213], brain injury [214]. Clinical trials in ALS [156, 215].

PI3K inhibitors	NVP-BEZ235 GSK2126458	Experimental trials in breast cancer [216].	LY 294002	Experimental trials in AD [217], PD [218].

Akt Inhibitors	Perifosine MK-2206 RX-0201 PBI-05204 GSK2141795	Clinical trials in advanced cancer [219, 220].	LY 294002	Experimental trials in AD [217], PD [218].

m-TOR	Everolimus Temsirolimus	Clinical use in pancreatic cancer [221], renal cancer [221]. Clinical trials in epithelial ovarian cancer/primary peritoneal cancer [222], Endometrial Cancer [223], glioblastoma [224], breast cancer [225], neuroendocrine tumours [226], lung cancer [227], bladder cancer [228], renal cancer [229, 230].	Rapamycin	Experimental trials in AD [231], PD [232], TBI [233, 234], SCI [235, 236], stroke [237].

Tau inhibitor	SG410	Experimental trials in pancreas cancer [238].	TRx-0014	Clinical use in AD [239, 240].

ERK1/2 kinase pathway	PD98059	Experimental trials in prostate cancer [241], lung cancer [242].	PD98059	Experimental trials in ICH [72, 92].

P38 kinase pathway	SB203580	Experimental trials in colon cancer [243].	SB203580 SB239063	Experimental trials in ICH [92], PD [244], stroke [245].

JNK kinase pathway	SP600125	Experimental trials in cancer cells [246, 247].	CEP-1347 Colostrinin SP600125	Experimental trials in ICH [72, 92], AD [248, 249], stroke [250], PD [248, 250].

4-Amino-5-(4-methylphenyl)-7-(t-butyl)pyrazolo(3,4-d)pyrimidine (PP1), 4-Amino-5-(4-chlorophenyl)-7(t-butyl)pyrazol(3,4-d)pyramide (PP2), 2-Amino-5,6-dihydro-6-methyl-4H-1,3-thiazine hydrochloride (AMT), 2,3-Dihydroxy-6-nitro-7-sulfamoyl-benzo [f]quinoxaline-2,3-dione (NBQX), Dl-2-amino-5-phosphonovaleric acid (AP5), Epigallocatechin-3-gallate (EGCG), Farnesylthiosalicylic acid (FTS), Methylthioinnium chloride (TRx-0014), 5-Methyl-10,11-dihydro-5H-dibenzo[a,d]cyclohepten-5,10-imine (MK 801), N-acytyl-L-cysteine (NAC), N(G)-nitro-l-arginine methyl ester (l-NAME), N(omega)-propyl-l-arginine (NPA), Phenyl-N-tert-butylnitrone (PBN), Superoxide dismutase (SOD).
